# Dobrava-Belgrade Virus in *Apodemus flavicollis* and *A. uralensis* Mice, Turkey

**DOI:** 10.3201/eid2001.121024

**Published:** 2014-01

**Authors:** I. Mehmet Ali Oktem, Yavuz Uyar, Ender Dincer, Aysegul Gozalan, Mathias Schlegel, Cahit Babur, Bekir Celebi, Mustafa Sozen, Ahmet Karatas, Nuri Kaan Ozkazanc, Ferhat Matur, Gulay Korukluoglu, Rainer G. Ulrich, Mustafa Ertek, Aykut Ozkul

**Affiliations:** Faculty of Medicine, Dokuz Eylul University, Izmir, Turkey (I.M.A. Oktem); Cerrahpasa Faculty of Medicine, Istanbul University, Istanbul, Turkey (Y. Uyar);; Ankara University, Ankara, Turkey (E. Dincer);; Refik Saydam National Public Health Agency, Ankara (A. Gozalan, C. Babur, B. Celebi, G. Korukluoglu, M. Ertek);; Friedrich-Loeffler-Institut, Greifswald-Insel Riems, Germany (M. Schlegel, R.G. Ulrich);; Karaelmas University, Zonguldak, Turkey (M. Sozen, F. Matur);; Nigde University, Nigde, Turkey (A. Karatas);; Bartin University, Bartin, Turkey (N.K. Ozkazanc);; Faculty of Veterinary Medicine, Ankara University, Ankara (A. Ozkul)

**Keywords:** Hantavirus, Dobrava-Belgrade virus, reservoir host, Turkey, Black Sea Coast, viruses

## Abstract

In 2009, human Dobrava-Belgrade virus (DOBV) infections were reported on the Black Sea coast of Turkey. Serologic and molecular studies of potential rodent reservoirs demonstrated DOBV infections in *Apodemus flavicollis* and *A. uralensis* mice. Phylogenetic analysis of DOBV strains showed their similarity to *A. flavicollis* mice–borne DOBV in Greece, Slovenia, and Slovakia.

The genus *Hantavirus,* family *Bunyaviridae,* contains human pathogenic viruses that cause hemorrhagic fever with renal syndrome (HFRS) and hantavirus cardiopulmonary syndrome (*1*). HFRS in Europe is caused mainly by Puumala virus and different genotypes of Dobrava-Belgrade virus (DOBV) (*2*). In Asia, Hantaan virus and Seoul virus cause most HFRS cases. Hantaviruses are enveloped viruses with a single-stranded 3-segmented RNA genome of negative polarity. The small (S), medium, and large genome segments encode the nucleocapsid protein, the glycoproteins Gn and Gc, and an RNA-dependent RNA polymerase, respectively.

Hantaviruses have been detected in various rodent, shrew, mole, and bat species (*3*). They are transmitted to humans by inhalation of aerosols that are contaminated with urine, feces, and saliva of infected reservoir hosts. The human pathogenic DOBV was first isolated from a yellow-necked field mouse (*Apodemus flavicollis*) and, subsequently, from a striped field mouse (*A. agrarius*) and a Caucasian wood mouse (*A. ponticus*) (*1*). The association of DOBV with these different *Apodemus* species seems to determine its human pathogenicity, with the *A. flavicollis–*associated genotype Dobrava being the most life threatening (*1*).

Few reports about hantavirus seroprevalence in human and rodent populations in Turkey occurred before 2009 (*4*,*5*). In February 2009, the first hantavirus outbreak among humans in this country was described in 2 provinces in the western Black Sea region (*6*; Figure 1). DOBV-reactive antibodies were reported for 7 of 200 patients who had renal symptoms in a region near the Aegean Sea (*7*). In 2010, DOBV RNA was detected by a nucleic acid test in urine from a person in Istanbul Province who was experiencing fatigue, diffuse pain, nausea, and vomiting (*8*). The reservoir host(s) and virus strain(s) causing human infections on the Black Sea coast of Turkey remained unknown.

**Figure 1:**
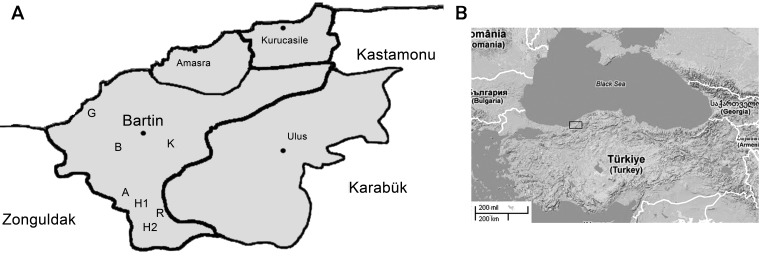
Regional map of Turkey showing the Bartin province (41°38′9′′N, 32°20′15′′E), where trapping of small mammals was conducted. Abbreviations indicate locations at which captures were performed: A, Akbas; B, Bogaz; H1, Hanyeri; H2, Hasankadi; G, Guzelcehisar; K, Kumluca; R, Region 49. Panel A corresponds to the region (Bartin Province) indicated by box in panel B.

## The Study

After the hantavirus outbreak in 2009, a National Hantavirus Study Group was founded in Turkey. Coordinated by this group, in June 2009, a total of 173 rodents and 2 shrews were collected from 7 sites in the rural area of Bartin Province in the western Black Sea Region, where suspected human hantavirus cases had been reported (Figure 1).

To determine the presence of DOBV in rodent tissue samples, we developed a single tube reverse transcription quantitative PCR (RT-qPCR) using the QuantiTect Probe RT-PCR kit (QIAGEN, Hilden, Germany) and novel primers and probe (Technical Appendix Table). For that purpose, spleen, liver, and lung samples from each animal were pooled and homogenized in sterile phosphate-buffered saline (pH 7.0). RNA extraction was performed by using a commercial viral nucleic acid isolation kit (Vivantis, Selangor Darul Ehsan, Malaysia). The reaction mixture (total 25 μL) of the RT-qPCR assay consisted of 1.5 μmol/L of each primer, 0.8 μmol/L of the probe, and 5 μL of RNA. Positive (100 copies of DOBV genotype Dobrava isolate(s) segment containing control plasmid) and negative control (ultrapure water) reactions were also included in each test. Thermal cycling was carried out in a RotorGene 6000 (QIAGEN) at 50°C for 30 min, 95°C for 15 min, then 45 cycles of 15 s at 94°C, 30 s at 60°C, and 1 min at 72°C.

The RT-qPCR revealed 23 DOBV RNA-positive rodents, including 10 *A. flavicollis mice*, 12 *A. uralensis* mice, and 1 *Rattus rattus* rat (Tables 1,2). The viral RNA load varied from 7.2 × 10^2^ to 4.6 × 10^6^ copies/mL. Most DOBV-positive rodents were from neighboring trapping sites in Akbas, Bogaz, and Kumluca Districts of Bartin Province (Figure 1). The reason hantavirus RNA could not be detected in bank voles (Table 1) might be because a DOBV-specific RT-qPCR was used that is unable to detect bank vole–associated PUUV.

**Table 1:** Detection rate of hantavirus-reactive IgM and IgG by ELISA, and hantavirus RNA by RT-qPCR in small mammals from different trapping sites, Turkey, 2009*

Location (map code/no. rodents)	No. positive animals/total no. animals														
	*Apodemus* spp.				*Myodes* spp.				*Rattus rattus*				Other†		
	IgM	IgG	RNA		IgM	IgG	RNA		IgM	IgG	RNA		IgM	IgG	RNA
Region 49 (R/11)	0/3	0/3	0/3		1/8	3/8	0/8		–	–	–		–	–	–
Akbaş (A/64)	0/39	1/39	8/39		2/18	14/18	0/18		–	–	–		0/5	0/5	1‡/5
Bogaz (B/20)	0/13	0/13	4/13		–	–	–		0/8	0/8	0/8		0/2	0/2	0/2
Guzelcehisar (G/17)	0/17	0/17	2/17		–	–	–		–	–	–		–	–	–
Hanyeri (H1/13)	0/8	0/8	2/8		0/5	3/5	0/5		–	–	–		–	–	–
Hasankadi (H2/33)	1/12	0/12	1/12		6/18	10/18	0/18		0/3	1/3§	1/3§		–	–	–
Kumluca (K/14)	1/14	4/14	5/14		–	–	–		–	–	–		–	–	–
Total no. positive/no. tested (%); N = 173	2/106 (1.9)	5/106 (4.7)	22/106 (20.7)		9/49 (18.3)	30/49 (61.2)	0/49		0/11	1/11 (9.1)	1/11(9.1)		0/7	0/7	1/7(14.2)

*RT-qPCR*,* reverse transcription quantitative PCR; –, not found. †*Pitymys* spp., *Microtus* spp*., Mus musculus, Crocidura suaveolens.*  ‡*Cytochrome b*–based genetic species determination was not performed. §Results from different animals.

**Table 2:** Serologic reactivity of 23 DOBV RT-qPCR–positive rodents by ELISA and IFA, Turkey, 2009*

Animal no.	Species (*cyt b*)†	Location/district‡	Hantavirus serology			
			ELISA			IFA
			IgM	IgG		IgG
94-09	Apodemus uralensis	Akbas	Neg	Neg		Neg
95-09	A. uralensis	Akbas	Neg	Neg		NS
121-09	A. flavicollis	Akbas	Neg	Neg		Neg
123-09	A. flavicollis	Akbas	Neg	Neg		Pos
124-09	A. uralensis	Akbas	Neg	Neg		BL
130-09	A. uralensis	Akbas	Neg	Neg		Neg
139-09	A. flavicollis	Akbas	Neg	Pos		Pos
142-09	A. flavicollis	Akbas	Neg	Pos		Neg
151-09	A. uralensis	Bogaz	Neg	Neg		Neg
162-09	A. uralensis	Bogaz	Neg	Neg		Neg
169-09	A. flavicollis	Bogaz	Neg	Pos		Neg
177-09	A. uralensis	Bogaz	Neg	Neg		Neg
178-09	A. uralensis	Guzelcehisar	Neg	Neg		NS
180-09	A. uralensis	Guzelcehisar	NS^§^	NS		NS
100-09	A. uralensis	Hanyeri	Neg	Neg		Neg
102-09	A. uralensis	Hanyeri	Neg	Neg		Neg
106-09	Rattus rattus	Hasankadi	Neg	Pos		Neg
25-09	A. flavicollis	Hasankadi	Pos	Neg		Neg
78-09	A. flavicollis	Kumluca	Neg	Pos		Pos
80-09	A. uralensis	Kumluca	Neg	Pos		Pos
81-09	A. flavicollis	Kumluca	Neg	Pos		Pos
134-09	A. flavicollis	Kumluca	Neg	Pos		Pos
137-09	A. flavicollis	Kumluca	Neg	Neg		Pos

*DoBV, Dobrava-Belgrade virus; RT-qPCR, reverse transcription quantitative PCR; IFA, immunofluorescence assay; NS, no serum sample available; BL, borderline. †C*ytochrome b*–based genetic species determination following a recently published protocol (*9*).  ‡In alphabetical order.

Serum samples from all 173 rodents were screened by a commercial enzyme immunoassay (Hantavirus DxSelect; Focus Diagnostic, Cypress, CA, USA) that used anti-mouse IgG and IgM conjugates (Sigma-Aldrich Chemie, Taufkirchen, Germany). The assay found that 11 (6.4%) and 36 (20.8%) animals contained hantavirus-reactive IgM and IgG, respectively (Table 1). Eight (4.6%) serum specimens were positive for both IgM and IgG, whereas 3 (1.7%) and 28 (16.2%) samples were positive for either IgM or IgG, respectively. The hantavirus seroprevalence varied between the different trapping sites and rodent species investigated (Table 1). Serologic status of the 23 RT-qPCR positive animals was proven by indirect immunofluorescence assay (IFA) as described by manufacturer (Euroimmune, Lübeck, Germany) except using fluorescein isothiocyanate (FITC)–labeled anti-mouse IgG conjugate (Sigma-Aldrich Chemie). The IgG ELISA results were confirmed for 5 samples, whereas 5 additional samples demonstrated a different reactivity in ELISA and IFA (Table 2).

All DOBV RT-qPCR positive samples were subjected to conventional S-segment RT-PCR, which produced an amplification product of 790 bp for sequence analysis. After denaturation of RNA at 70°C for 5 min, cDNA was synthesized by using Moloney murine leukemia virus RT and random hexamers (MBI Fermentas, Vilnius, Lithuania), by incubating at 25°C for 10 min and thereafter at 37°C for 1 h. PCR amplification was carried out by adding 3 μL of cDNA into a reaction mix containing 75 mmol/L Tris-HCl (pH 8.8), 20 mmol/L NH_4_(SO_4_)_2_, 2.4 mmol/L MgCl_2_, 10 pmol of each primer, 0.2 mmol/L dNTP, and 5U Taq DNA polymerase (MBI Fermentas). The initial denaturation for 6 min at 94°C, was followed by 40 cycles each at 94°C for 1 min, 52°C for 1 min, 72°C for 2 min, and a final extension at 50°C for 1 min and 72°C for 10 min. Analysis by agarose gel electrophoresis revealed amplification products of the expected size for 21 samples. The PCR products were purified by using PCR/Gel purification kit (GeneMark Technology Co., Taichung City, Taiwan) and cloned into pJet1.2 blunt (MBI Fermentas). Plasmid DNA was directly subjected to sequencing in CEQ 8000 Genetic Analyzer (Beckmann Coulter, Brea, CA, USA) using the Dye Termination Cycle Sequencing Kit (Beckmann Coulter).

**Figure 2:**
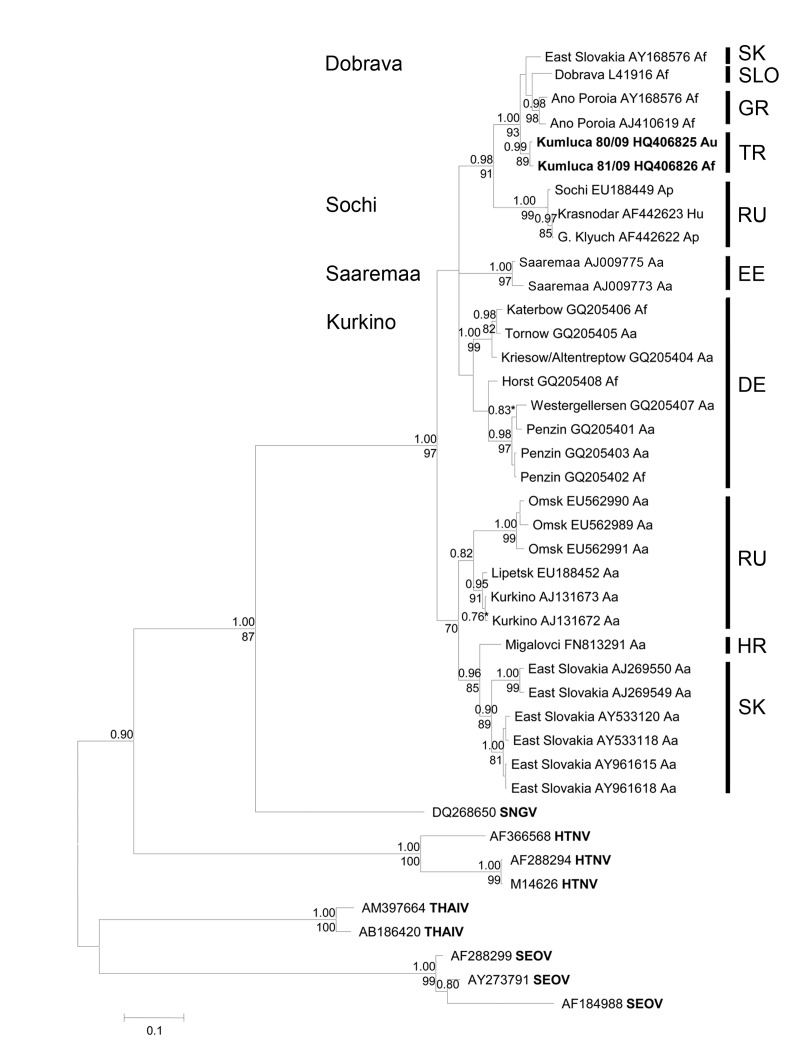
Bayesian phylogenetic tree, based on an alignment of 450-nt long region of the small segment from various Dobrava-Belgrade virus lineages and other Murinae-associated hantaviruses. Posterior probabilities for Bayesian analysis are given under the branches and bootstrap values above the branches. Values lower than <0.7% and <70% are not shown. The sequences were aligned with ClustalW included in the BioEdit software package version 2.1 (http://www.mbio.ncsu.edu/bioedit/page2.html). The phylogenetic analyses were performed by using MrBayes 3.1.2 with Bayesian Metropolis-Hastings Markov Chain Monte Carlo (MCMC) tree-sampling methods based on 2 MCMC runs consisting of 4 chains of 2,000,000 with a burn-in of 25% and second by maximum-likelihood bootstrap analysis with 1,000 pseudoreplicates using MEGA5 (www.megasoftware.net). The Hasegawa-Kishino-Yano model with a discrete gamma distribution, to model evolutionary rate differences among sites (2 categories [+G, parameter = 0.8874]) according to jModeltest (http://code.google.com/p/jmodeltest2/) was used. Af, *Apodemus flavicollis*; Au, *A. uralensis*; Ap, *A. ponticus*; Hu, human; Aa, *A. agrarius*; SNGV, Sangassou virus; HTNV, Hantaan virus; THAIV, Thailand virus; SEOV, Seoul virus; SK, Slovakia; SLO, Slovenia; GR, Greece; TR, Turkey; RU, Russia; EE, Estonia (island Saaremaa); DE, Germany; HR, Croatia. Scale bar indicates number of nucleotide substitutions per site.

The nucleotide sequence identity among the obtained 21 S-segment sequences (GenBank accession nos. KF615886–KF615906) was very high, reaching 99.2 %. For phylogenetic analysis 2 novel DOBV strains found in *A. flavicollis* (Table 1, number 81/09; accession no. HQ406826) and *A. uralensis* (Table 1, number 80/09; accession no. HQ406825) were selected that showed a nucleotide sequence identity of 99.1%, whereas the amino acid sequence identity of the corresponding part of the nucleocapsid protein was 98.7%. A phylogenetic analysis of these 2 *Apodemus*–derived DOBV sequences from Turkey confirmed their strong similarity to *A. flavicollis*–associated DOBV genotype Dobrava sequences from Greece, Slovenia, and Slovakia (Figure 2). The sequence divergence of the 2 sequences from Turkey to other genotype Dobrava sequences was found to be 3.8% at nucleotide level (0.7% at amino acid level). The sequence divergence to DOBV sequences from *A. ponticus* and *A. agrarius* mice was found to be much higher, up to 15.2% and 6%, respectively.

## Conclusions

Human infections with members of the *A. flavicollis*–associated DOBV lineage have been described in Slovenia, Greece, Serbia, Montenegro, the Czech Republic, and Hungary (*2*). The current study of rodents in Turkey identified DOBV genotype Dobrava (DOBV-Af) as a potential causative agent of the hantavirus outbreak on the Black Sea coast of Turkey. This conclusion is in agreement with previous findings in patients from this region (*6*,*8*,*10*).

Notably, this study demonstrated closely related DOBV sequences in *A. flavicollis* and *A. uralensis* mice and a high DOBV prevalence in both species. This observation might indicate multiple DOBV spillover to *A. uralensis* mice. In addition, we found a single spillover infection here in rats (*R. rattus*). Similarly, spillover infections of DOBV genotype Dobrava (DOBV-Af) in *Mus musculus* and *A. sylvaticus* mice and of DOBV genotype Kurkino (DOBV-Aa) in *A. flavicollis* mice have been reported (*11*,*12*). Alternatively, the frequent detection of DOBV-specific nucleic acid in *A. uralensis* mice may indicate that this rodent species functions as a reservoir. Previously, in a study in Czech Republic *A. uralensis* mice were found to contain hantavirus antigen (*13*). These findings underline the current problem of identifying hantavirus reservoirs (*14*) as was recently also observed for European Tula virus (*15*). The detection of hantavirus-reactive antibodies and the absence of hantavirus RNA in *Myodes* species might be explained by DOBV spillover infections or, alternatively, by the presence of another hantavirus not detected by the RT-qPCR assay used here. Therefore, future studies on sympatrically occurring *Apodemus* species at multiple sites in Turkey along the distribution range of *A. uralensis* mice need to confirm whether these rodents have a role as a potential reservoir host of DOBV. In addition, a comprehensive study in different rodent and other small mammal species should determine whether hantaviruses other than DOBV are present.

Technical Appendix List of oligonucleotides used for reverse transcription quantitative PCR and sequencing of small segments of Dobrava-Belgrade virus strains, Turkey, 2009.

## References

[1] Krüger DH, Schonrich G, Klempa B. Human pathogenic hantaviruses and prevention of infection. Hum Vaccin. 2011;7:685–93 . 10.4161/hv.7.6.15197 21508676 PMC3219076

[2] Klempa B, Avsic-Zupanc T, Clement J, Dzagurova TK, Henttonen H, Heyman P, Complex evolution and epidemiology of Dobrava-Belgrade hantavirus: definition of genotypes and their characteristics. Arch Virol. 2013;158:521–9 . 10.1007/s00705-012-1514-5 23090188 PMC3586401

[3] Guo WP, Lin XD, Wang W, Tian JH, Cong ML, Zhang HL, Phylogeny and origins of hantaviruses harbored by bats, insectivores, and rodents. PLoS Pathog. 2013;9:e1003159 . 10.1371/journal.ppat.1003159 23408889 PMC3567184

[4] Laakkonen J, Kallio-Kokko H, Öktem MA, Blasdell K, Plyusnina A, Niemimaa J, Serological survey for viral pathogens in Turkish rodents. J Wildl Dis. 2006;42:672–6 . 10.7589/0090-3558-42.3.672 17092901

[5] Kavukcu S, Turkmen M, Salman S, Soylu A, Camsari T. What is the risk of nephropathy associated with hantavirus in Aegean region? J Turkish Nephrol Assoc. 1997;3–4:131.

[6] Ertek M, Buzgan T. An outbreak caused by hantavirus in the Black Sea region of Turkey, January–May 2009. Euro Surveill. 2009;14:19214 . 19460288 10.2807/ese.14.20.19214-en

[7] Oktem MA. Hantavirus and tick-borne encephalitis infections [in Turkish]. Ankem Derg. 2009;23(Suppl 2):245–8.

[8] Oncul O, Atalay Y, Onem Y, Turhan V, Acar A, Uyar Y, Hantavirus infection in Istanbul, Turkey. Emerg Infect Dis. 2011;17:303–4 . 10.3201/eid1702.100663 21291612 PMC3204758

[9] Schlegel M, Ali HS, Stieger N, Groschup MH, Wolf R, Ulrich RG. Molecular identification of small mammal species using novel cytochrome b gene–derived degenerated primers. Biochem Genet. 2012;50:440–7. 10.1007/s10528-011-9487-8 22193288

[10] Heyman P, Cochez C, Korukluoglu G, Gozalan A, Uyar Y, Lundkvist A. Bridging continents; Hantaviruses of Europe and Asia Minora; bridging continents. Hantaviruses of Europe and Asia Minor. Turk Hij Den Biyol Derg. 2011;68:41–8.

[11] Weidmann M, Schmidt P, Vackova M, Krivanec K, Munclinger P, Hufert FT. Identification of genetic evidence for Dobrava virus spillover in rodents by nested reverse transcription (RT)-PCR and TaqMan RT-PCR. J Clin Microbiol. 2005;43:808–12. 10.1128/JCM.43.2.808-812.2005 15695684 PMC548048

[12] Schlegel M, Klempa B, Auste B, Bemmann M, Schmidt-Chanasit J, Buchner T, Dobrava-Belgrade virus spillover infections, Germany. Emerg Infect Dis. 2009;15:2017–20. 10.3201/eid1512.090923 19961690 PMC3044545

[13] Heroldová M, Pejcoch M, Bryja J, Janova E, Suchomel J, Tkadlec E. Tula virus in populations of small terrestrial mammals in a rural landscape. Vector Borne Zoonotic Dis. 2010;10:599–603. 10.1089/vbz.2009.0211 20420534

[14] Hjelle B, Yates T. Modeling hantavirus maintenance and transmission in rodent communities. Curr Top Microbiol Immunol. 2001;256:77–90. 10.1007/978-3-642-56753-7_5 11217407

[15] Schlegel M, Kindler E, Essbauer SS, Wolf R, Thiel J, Groschup MH, Tula virus infections in the Eurasian water vole in Central Europe. Vector Borne Zoonotic Dis. 2012;12:503–13. 10.1089/vbz.2011.0784 22225425

