# Supplementary material for: Dobrava-Belgrade Virus in Apodemus flavicollis and A. uralensis Mice, Turkey
Source: Emerg Infect Dis. 2014 Jan;20(1):121–5. doi: 10.3201/eid2001.121024 (PMC3887490; doi:10.3201/eid2001.121024)
Supplement: Technical Appendix — List of oligonucleotides used for reverse transcription quantitative PCR and sequencing of small segments of Dobrava-Belgrade virus strains, Turkey, 2009. [file 12-1024-Techapp-s1.pdf]

Article DOI: http://dx.doi.org/10.3201/eid2001.121024

Dobrava-Belgrade Virus in *Apodemus flavicollis* and *A. uralensis* Mice, Turkey

Technical Appendix

Technical Appendix Table. List of oligonucleotides used for reverse transcription quantitative PCR and sequencing of small segments of Dobrava-Belgrade virus strains, Turkey, 2009

Name*	Sequence (5'→3')	Purpose of use	Expected product size (in bp)
Dob/S/729/F	TGGACAGAGCGGGTTGARG		
Dob/S/795/R	ACGTTGGAGATGGCTCAGATAGTA	RT-qPCR	67
Dob/S/748/Pr	FAM-AATGGCTTGACCTCCCGTGCAA-TAMRA		
Dob/S/650/F	ATACCCAGCCCAGGTTAAGG		
Dob/S/1440/R	CCAACCCAACCCCTAGTGTA	Sequencing	790

*Serotype/segment/nucleotide number/direction of primer or probe. These novel primers and probe were designed on the basis of an alignment of Dobrava-Balgrade virus small segment sequences from neighboring geographic regions. F, forward; R, reverse; Pr, probe
